# Physiological synaptic signals initiate sequential spikes at soma of cortical pyramidal neurons

**DOI:** 10.1186/1756-6606-4-19

**Published:** 2011-05-08

**Authors:** Rongjing Ge, Hao Qian, Jin-Hui Wang

**Affiliations:** 1 State Key Lab for Brain and Cognitive Sciences, Institute of Biophysics, Chinese Academy of Sciences, Beijing, China 100101

**Keywords:** action potential, soma, axon, refractory period, sodium channels

## Abstract

The neurons in the brain produce sequential spikes as the digital codes whose various patterns manage well-organized cognitions and behaviors. A source for the physiologically integrated synaptic signals to initiate digital spikes remains unknown, which we studied at pyramidal neurons of cortical slices. In dual recordings from the soma vs. axon, the signals recorded *in vivo *induce somatic spikes with higher capacity, which is associated with lower somatic thresholds and shorter refractory periods mediated by voltage-gated sodium channels. The introduction of these parameters from the soma and axon into *NEURON *model simulates sequential spikes being somatic in origin. Physiological signals integrated from synaptic inputs primarily trigger the soma to encode neuronal digital spikes.

## Introduction

The neurons are one of basic units to fulfill the brain functions, and their events are executed at different subcellular compartments, such as the reception of synaptic inputs, the integration of these synaptic signals, the production of action potentials and the secretion of neurotransmitters [1,2]. In terms of the sources for firing action potentials, the current belief is that action potentials are generated at axon hillock [3-11]. In these studies, short-time square pulses are given and a single spike is induced. However, the regulations and mechanisms for the physiological signals integrated from synaptic inputs to trigger the spikes remains unknown.

**Figure 1:**
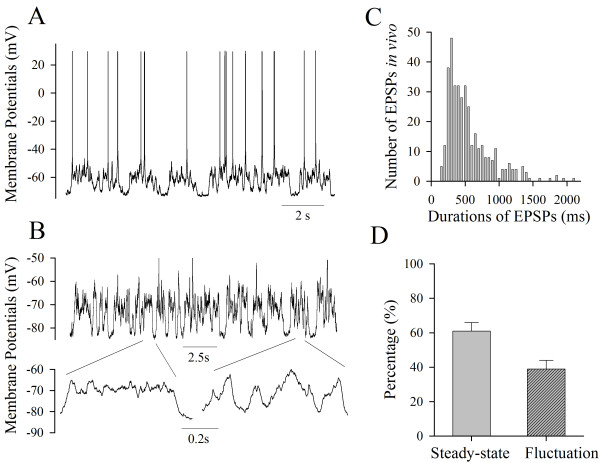
***In vivo *depolarization signals for spike initiation and subthreshold are long-time in nature**. **A) **The integrated synaptic signals induce spikes. **B) **shows the integrated synaptic signals at subthreshold level (top panel) and the expanded waveforms (bottoms), which appear steady-state pattern (square pulse) and fluctuation one (cosine). **C) **shows number of EPSPs *in vivo *vs. signal durations, which fall into a range of 50~1600 ms. **D) **illustrates the percentages for steady-state pattern and fluctuation one analyzed from total *in vivo *signals (n = 11 neurons).

The neurons integrate the signals from numerous synapses and produce sequential spikes as the digital codes to carry various messages under the physiological conditions [12,13]. These integrated signals *in vivo *are long-time in nature, and their depolarization pulses induce sequential spikes [14-18] and Figure 1). A source for these *in vivo *signals to initiate sequential spikes has not been documented, which we have investigated at cortical pyramidal neurons by dual- recording their soma and axonal bleb simultaneously.

## Results

### Integrated synaptic signals are long-time pulses in patterns of steady-state and fluctuation

The physiological sources of firing action potentials are ideally identified by using *in vivo *signals, which has not been documented yet. In order to address this issue, we have analyzed these signals that were intracellularly recorded from cortical pyramidal neurons in living mice.

*In vivo *signals including those inducing sequential spikes (Figure 1A) and subthreshold pulses (Figure 1B) appear long time. Figure 1C illustrates that these depolarization pulses integrated *in vivo *fall into a range of 50~1600 ms in their durations. These *in vivo *signals are generally classified into steady-state pulses (an extended waveform in left panel of Figure 1B) and fluctuation ones (in right). The former is similar to direct-current pulses used to induce spikes in the most of electrophysiological experiments, and the latter is simulated as a cosine model [19]. The percentages of steady-state forms and fluctuation ones in these *in vivo *signals are approximately 61 ± 5% and 39 ± 5%, respectively (Figure 1D, n = 11 cells). Therefore, the physiological signals to induce sequential spikes are long-time depolarization pulses, which we used to identify the sources of sequential spikes.

### Physiological synaptic signals induce sequential spikes more efficiently at the soma than axon

**Figure 2:**
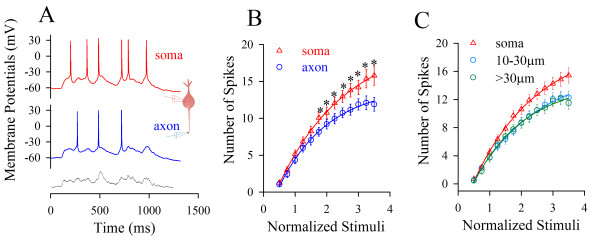
***In vivo *signals initiate sequential spikes preferentially at the soma of cortical pyramidal cells**. **A) **An *in vivo *pulse (black trace at bottom, >1000 ms) evokes more spikes at the soma (red trace) than at axon (blue) in dual recording (inset). **B) **shows spike number vs. normalized pulses *in vivo *at the soma (triangle symbols) and axon (circles; n = 19; asterisks, p < 0.05). **C) **illustrates input-output curves for the soma (red symbols) and different axonal fragments in 10~30 μm (blue) and >30 μm (green) away from the soma.

In theory, no matter what the axon or soma is a primary site to encode sequential spikes, it should have the higher ability to convert analogue input signals into digital spikes, i.e., more efficient input- output. The soma and axonal bleb (20~45 μm away from the soma) of identical pyramidal neurons in neocortical slices were recorded simultaneously in whole-cell current-clamp (inset in Figure 2A). The i*n vivo *signals (bottom trace in Figure 2A) in various intensities were injected into these two locations, respectively, to assess their input-output couplings. These long-time pulses induced more spikes at the soma (red trace in Figure 2A) than the axon (blue). Figure 2B shows spike number vs. normalized pulses *in vivo *at the soma (red triangles) and axon (blue circles, n = 19), in which somatic input-output curve is on the top side of axonal one. The facts that somatic spike thresholds are lower and identical stimuli induce more spikes at the soma indicate a somatic origin of firing sequential spikes.

If the soma encodes spikes, it should have the highest ability of firing spikes in response to inputs, like a principle that sinoatrial node in the highest rate controls heart beat. Figure 2C shows input-output curves at the soma (red symbols, n = 27) and axonal segments in 10~30 μm (axon hillock; blue, n = 15) and >30 μm (green, n = 12) away from the soma i.e., a decreasing trend in the ability of firing spikes from the soma to the axon. This result indicates that the soma is more sensitive to long-time signals and dominantly produces spikes. The physiological *in vivo *signals induce sequential spikes primarily at the soma of cortical pyramidal neurons.

### Latencies between somatic spikes and axonal ones favor a somatic origin of spike initiation

**Figure 3:**
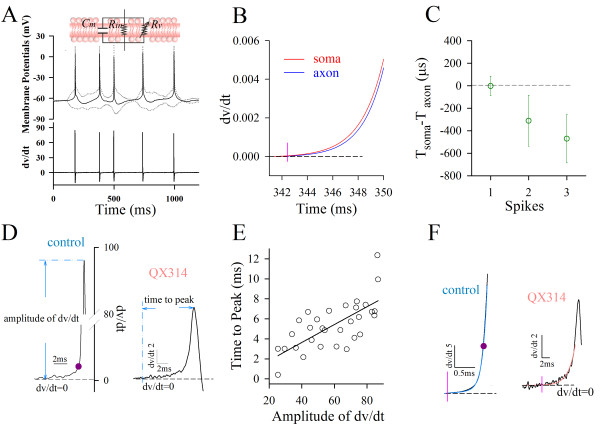
**Latencies between somatic spikes and axonal ones favor somatic origins of sequential spikes**. **A) **Top panel shows an electrical circuit for cellular membrane, Cm, membrane capacitance; Rin input resistance and Rv, voltage-gated conductance. Middle panel illustrates sequential spikes (black line) subtracted from the responses (gray dot-line) by depolarization and hyperpolarization. Bottom shows the derivative of spike potentials with respect to time (*dv/dt*). **B) **shows the expanded waveforms of dv/dt vs. time for a somatic spike (red trace) and axonal one (blue). A vertical line shows a location of spike initiation, which is defined as a time point of minimal *dv/dt *but larger than zero. **C) **illustrates latencies between somatic spikes and axonal ones (ΔT = Tsoma-Taxon) versus spikes. **D) **shows the measurement in the amplitudes of spike *dv/dt *and the time of minimal *dv/dt *to peak in the intracellular use of QX-314 (0.5 mM). **E) **shows the proportional correlation between the amplitudes of spike *dv/dt *and the time of minimal *dv/dt *to peak (36 spikes from three cells) in the partial inactivation of VGSCs. **F) **The rising phase of spikes is better fitted into two exponentials under the control (r^2 ^= 0.99), and a single exponential (r^2 ^= 0.99) under QX-314 application.

The initiation of spikes was defined at a time point of minimal *dv/dt *but larger than zero (Figure 3A~B and Methods). Latencies between somatic spikes and axonal ones (ΔT) were calculated. Figure 3C shows that ΔT values for spikes 1~3 are -2.5 ± 87, -311 ± 226.5 and -471 ± 215.8 μs (n = 20), respectively. In spite of big variation for ΔT values, sequential spikes recorded at the soma appear ahead of those at the axon.

It is noteworthy that we validated the approach used to localize the time point of spike initiation in Figure 3A~B. If the time point of minimal *dv/dt *is a good measurement to locate spike onset mediated by voltage-gated sodium channels (VGSC), it should be associated with spikes, and the manipulation of VGSCs should affect this point and spikes proportionally. In other words, if VGSCs are partially blocked, we should see the proportional changes of its time phases with spike amplitudes, as well as observe the presence of this point and the spikes with a loss of the initiation sites that were previously defined [20-24].

Based on analyzing the amplitudes of spike *dv/dt *and the time of minimal *dv/dt *to its peak in the presence of QX-314 that is an inhibitor of VGSCs (0.5 mM intracellularly in Figure 3D), we observed the proportional correlation between these two parameters (36 spikes from 3 cells in Figure 3E) under the condition of partial VGSCs' inactivation. Moreover, spike initiation defined previously (purple dots in 3D and 3F) is located between minimal *dv/dt *site and spike fast-rising phase, which divides the spike rising-phase into two components exponentially (blue trace in 3F). Figure 3F shows that *dv/dt *values of spike rising-phase are better fitted into two exponentials under control (r^2 ^= 0.99) and an exponential (r^2 ^= 0.99) under partial VGSC inactivation. A loss of spike-onset site previously defined indicates that the previous site is not always associated with spikes. Taken these data together, we conclude that the minimal *dv/dt *site is better for representing spike initiation.

### VGSC-mediated spike thresholds and refractory periods are lower at the soma than axon

**Figure 4:**
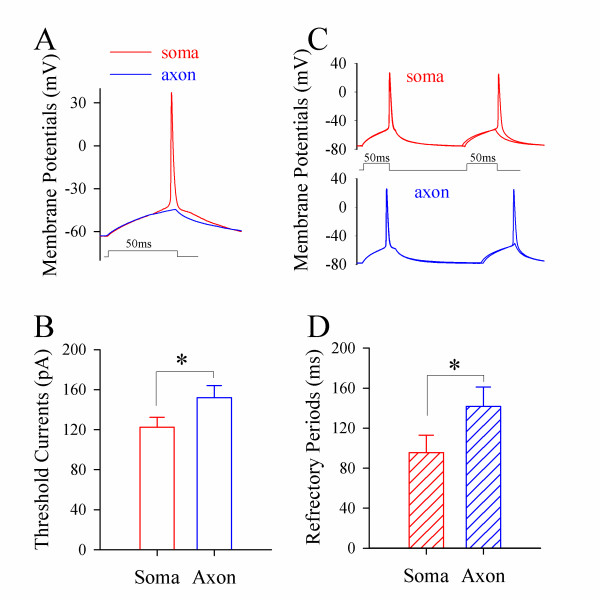
**Spike thresholds and refractory periods induced by steady-state pulses (50 ms) are lower at the soma than the axon, which simulates somatic spikes in origin**. **A) **Somatic spike thresholds appear lower at the soma (red trace) than axon (blue). **B) **shows somatic spike threshold (red bar) vs. axonal one (blue; asterisk, p < 0.05; n = 12). **C) **Somatic spike refractory periods appear shorter at the soma (red trace) than axon (blue). **D) **Somatic spike RPs (red bar) are shorter than axonal ones (blue; p < 0.05; n = 9).

In terms of mechanisms for physiological signals to primarily induce somatic spikes, we propose that long-time depolarization makes somatic spike thresholds and refractory periods lower than axonal ones. Thresholds and refractory periods were measured by injecting depolarization pulses (50 ms, a minimal duration of *in vivo *signals in Figure 1, and Methods) into the soma and axonal bleb of identical neurons, respectively [14,25]. A pulse induced a spike at the soma (red trace in Figure 4A) but not the axon (blue). Figure 4B illustrates that somatic threshold (122.5 ± 10 pA, red bar) is lower than axonal one (152 ± 12 pA, blue; p < 0.05; n = 12).

Moreover, paired-pulses (50 ms for their duration) were used to measure the refractory periods of somatic spikes (red traces in Figure 4C) and axonal ones (blues). Figure 4D shows that somatic refractory periods (red bar) and axonal ones (blue) are 95.5 ± 17.6 and 141.7 ± 19.3 ms, respectively (p < 0.05; n = 9). When long-time depolarization signals are integrated at pyramidal cells, somatic spike thresholds and refractory periods are lower than axonal ones, which make sequential spikes primarily induced at cell body.

**Figure 5:**
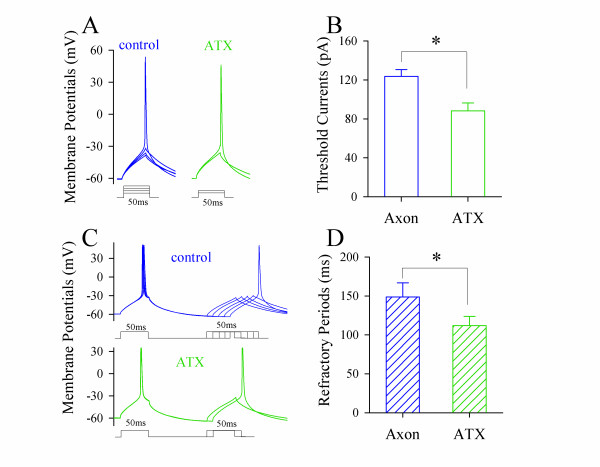
**The prevention of axonal VGSC inactivation by ATX decreases axonal spike thresholds and shortens refractory periods**. **A) **shows the waves of axonal spike thresholds induced by depolarization pulses (50 ms) before and after ATX application (5 μM, green trace). **B) **shows the averaged values of axonal spike thresholds before and after ATX application. **C) **shows the waveforms of axonal spike refractory periods induced by depolarization pulses (50 ms) before and after ATX application (5 μM, green trace). **D) **shows the averaged values of axonal spike refractory periods before and after ATX application.

To the mechanism underlying lower somatic thresholds and refractory periods by long-time pulse, we propose that long-time depolarization partially inactivates axonal voltage-gated sodium channels (VGSC). If it is a case, we should see the prevention of VGSC inactivation lowers axonal thresholds and refractory periods. Anemone toxin (ATX) that attenuates VGSC inactivation [26] was selected to prevent axonal VGSC inactivation. We measured thresholds and refractory periods of axonal spikes induced by depolarization pulses (50 ms) during applying 5 μM ATX onto axonal blebs. ATX appears to reduce axonal spike thresholds (green traces in Figure 5A) and refractory periods (green in Figure 5B). Figure 5C~D illustrate spike thresholds and refractory periods before (blue bars) and after ATX application (greens; p < 0.05, n = 9), respectively. The results indicate that long-time depolarization increases axonal thresholds and refractory periods through inactivating local VGSCs.

### Lower thresholds and refractory periods at the soma simulate a somatic origin of spikes

**Figure 6:**
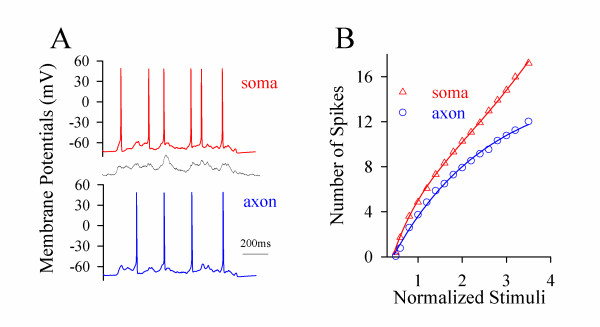
**Computational simulation based on spike thresholds and refractory periods at the soma and axon favors the sequential spikes being somatic in origin**. **A) **The number of spikes in computational simulation is higher at the soma (red trace) than the axon (blue) induced by *in vivo *signal (middle). **B) **illustrates the number of spikes vs. the intensity of stimulus pulses at the soma (red triangle symbols) and the axon (blue circles).

The results above indicate that long-time signals mainly inactivate axon's VGSCs and lead to the high values of spike thresholds and refractory periods. As a result, physiological signals initiate spikes being somatic in origin. To strengthen this point, we introduced the factors from the axon and soma into *NEURON *[27,28]. *In vivo *signals induced more simulated spikes at the soma (red trace in Figure 6AE) than the axon (blue). The thresholds of spikes are lower and the number of spikes by identical stimuli is higher at the soma (red symbols in Figure 6B) than the axon (blue). The result from computational simulation is consistent with those from experiments (Figure 2).

## Discussion

Long-time *in vivo *signals induce sequential spikes at the soma of cortical pyramidal neurons (Figure 2). In terms of the mechanism, these pre-depolarization signals mainly inactivate axonal VGSCs (Figures 4~5), such that somatic thresholds and refractory periods are lower (Figure 4), and the sequential spikes are somatic in origin (Figure 6). Our data from the experiments and computational simulation favor a somatic source for physiological signals to induce digital spikes, which updates the knowledge that an action potential is only initiated at the axonal hillock. Our data also supports a hypothesis that spikes may be generated on dendrites or soma in other types of neurons [29-36].

Short-time pulses initiate a single spike at axon hillock [3,6,8], which is supported by low thresholds and high density of VGSCs at this segment [4,5,9-11,37-48], and axonal spikes ahead of somatic ones [20-24]. The major reasons for this belief versus our findings include the followings. Previous studies used short-time pulses (less than 5 ms) to induce a single spike, but we applied long-time depolarization signals that fall into the range of the durations of integrated synaptic inputs (Figure 1C) to induce sequential spikes. More importantly, the pulses used in our studies are the *in vivo *signals (Figures 1,2,3), which should be more physiological.

We do observe that short-time pulses make axonal threshold and refractory period low, and evoke an axonal spike. However, the signals *in vivo *are above 50 ms (Figure 1), and are integrated from unitary synaptic events that last for longer than 20 ms [13,14]. Long-time pulses make somatic thresholds and refractory periods to be lower than axonal ones (Figure 4), such that physiological signals induce sequential spikes being somatic in origin. In this regard, there may be a plasticity of spike-onset location that depends on the patterns of synaptic inputs, as we have observed.

VGSC densities are high at the remote side of axonal hillock such that computational simulations show an axonal origin of spike initiation [4,9,10,45,46]. However, the number of functional VGSCs is not higher at axonal hillock [49]. Axonal VGSCs may be easily inactivated and less reactivated by long-time pulses (Figures 4~5). Therefore, the initiation of sequential spikes depends on the functional states of VGSCs, instead of their higher densities. One could argue the results that sodium imaging indicates high functional VGSCs' density at AIS [20,22,50]. As somatic volume is much larger than axonal one, the quantity of Na^+ ^influx during firing spikes is buffered substantially at the soma. Less change in the somatic Na^+ ^signal may not reflect a low level of functional VGSCs and a slow initiation of spikes at the soma.

A measurement of latencies between axonal spikes and somatic ones indicates the origin of spike initiation at AIS [20-24]. In such studies, the effect of passive membrane property on measuring temporal signals was present, and spike initiation was defined at various time points. With reducing the effect of membrane property and defining minimal *dv/dt *as a site of spike initiation that is always associated with spikes (Figure 3), we found that the latency between somatic spikes and axonal ones favors somatic spikes in origin.

What is physiological significance for higher dense VGSCs at axonal hillock? It may facilitate somatic spikes propagating toward axonal terminals [14]. Moreover, the nearby end of AIS is innervated by GABA synapses [51] and has less VGSCs [9,10,43]. The higher dense VGSCs at remote AIS, while GABA synapses induce a hyperpolarization, compensate loss of signals at nearby AIS, a homeostasis between subcellular compartments [12].

What is the physiological significance for the somatic source of sequential spikes? If the soma integrates input signals to initiate spikes at the axon, these integrated signals should be propagated to the axon. That long-time depolarization signals inactivate axonal VGSCs and GABAergic synapses at AIS shut these signals will weaken this propagation. To prevent any potential dissociation between the integration of synaptic signals and the conversion of them into digital spikes, these two processes are better fulfilled in a single unit, i.e., the soma.

## Materials and methods

### Brain slices

Cortical slices (300 μm) were prepared from FVB-Tg(GadGFP)45704Swn/J mice. The mice in postnatal days 15-20 were anesthetized by injecting chloral hydrate (300 mg/kg) and decapitated with a guillotine. The slices were cut with a Vibratome in the modified and oxygenized (95% O_2_/5% CO_2_) artificial cerebrospinal fluid (mM: 124 NaCl, 3 KCl, 1.2 NaH_2_PO_4_, 26 NaHCO_3_, 0.5 CaCl_2_, 5 MgSO_4_, 10 dextrose and 5 HEPES; pH 7.35) at 4°C, and then were held in the normal oxygenated ACSF (mM: 126 NaCl, 2.5 KCl, 1.2 NaH_2_PO_4_, 26 NaHCO_3_, 2.0 CaCl_2_, 2.0 MgSO_4 _and 25 dextrose; pH 7.35) 35°C for 1 hour before the experiments. A slice was transferred to a submersion chamber (Warner RC-26G) that was perfused with normal ACSF for electrophysiological experiments [52,53]. The entire procedures were approved by Institutional Animal Care Unit Committee in Administration Office for Laboratory Animals, Beijing China (B10831).

### Dual recording

The soma and axonal bleb of identical pyramidal neurons in layers IV-V of cerebral cortex were simultaneously recorded (MultiClapm-700B, Axon Instrument Inc. USA) under a fluorescent and DIC microscope (Nikon FN-E600; [14]. Electrical signals were inputted into pClamp-10 with 50 kHz sampling rate. In whole-cell recording, action potentials were induced by the signals recorded intracellularly *in vivo*. The judgment for recording two sites from an identical neuron is based on the synchronous presence of direct and corresponding electrical signals. Transient capacitance was compensated. Output bandwidth was 3 kHz. Pipette solution contains (mM) 150 K-gluconate, 5 NaCl, 0.4 EGTA, 4 Mg-ATP, 0.5 Tris- GTP, 4 Na-phosphocreatine and 10 HEPES (pH 7.4 adjusted by 2M KOH). The osmolarity of pipette solution was 295-305 mOsmol. The pipette resistance was 10-15 MΩ.

Neuronal intrinsic properties include spike thresholds (Vts) and refractory periods (RP). Vts were measured by depolarization pulses. RPs were measured by injecting two pulses (5% above threshold) into neurons after each spike under current-clamp, in which inter-pulse intervals were adjusted [12,13,25,54,55]. The duration of pulses was 50 ms, the minimal time period of *in vivo *signals (Figure 1C)

Latencies between axonal spikes and somatic ones, used to judge spike initiation, were measured based on the following thoughts. Elements in an electrical circuit of cell membrane includes voltage- gated conductance (Rv) for the generation of active signals, such as action potentials and synaptic signals, as well as passive membrane properties (input resistance, Rin; membrane capacitance, Cm; inset in Figure 3A). We ruled out the effects of Rin and Cm on the analyses of temporal signals via subtracting the responses (gray lines in 3A) evoked by depolarization and hyperpolarization in the same intensities, such that spike potentials (black line in Figure 3A) were mediated by voltage-gated channels. The derivative of somatic and axonal spike potentials vs. time (*dv/dt*) was calculated. The site of spike initiation was defined as a time point with a minimal *dv/dt *but larger than zero (Figure 3B), which accurately represents the locus of spike initiation in the comparison with the peak, 50% rising phase or initial phase (onset point) of spikes [20-24,50,56-58]. Latencies between somatic spikes and axonal ones were the time difference of their initiation (ΔT = Tsoma-Taxon).

### *In vivo *recording

Intracellular recordings with sharp electrodes were done at cortical pyramidal neurons from mice *in vivo *that were anesthetized by injecting chloral hydrate (300 mg/kg). Electrical signals were recorded by an AxonClamp-2B and sampled by pClamp-9 with 50 kHz sampling rate (Axon Instrument Inc, USA). Electrodes were filled with 2M potassium acetate and their resistances were 50~70 MΩ. The data were analyzed if resting membrane potentials were -65 mV, and action potentials showed overshot [14].

### Computational Model

The values of axonal and somatic Vts and ARP (Figure 3) were introduced into the axon and soma in NEURON (v7.0), respectively, to examine spikes' initiation. Other factors of VGSCs were based on Hodgkin-Huxley kinetics and current reports [9-11]. VGSCs' distributions in two compartments were based on the references [4,5,9-11]. VGSCs' reversal potential was 50 mV. For potassium channels, high-voltage-activated K^+ ^channels (Kv) and a slowly-activated/non-inactivated M-type K^+ ^channels (Km) were added into the modeling. To have the initiation of sequential spikes, the densities of Kv and Km were 50 and 100 pS/cm^2 ^in the two compartments, respectively. Reversal potential for Kv was -77 mV. In addition, cylinder axon was calculated based on 1.6 μm in diameter and 70 μm in length, and the soma was 30 μm in diameter. Their passive electrical properties include *C*_m _= 1 μF/cm^2^, *R*_m _= 15000 Ω/cm^2 ^and *R*_i _= 100 Ω/cm. Resting membrane potentials were -71 mV. The simulations were run under 37°C. The time step was 0.025 ms.

### Data analyses

The data were analyzed if the soma and axon had resting membrane potentials negatively more than -63 mV and action potentials above 95 mV. Criteria for the acceptation of each experiment also included less than 5% changes in resting membrane potential, spike magnitude, input and seal resistance during each experiment. The values of Vts and RP are presented as mean ± SE. The comparisons between groups are done by paired t-test.

## Competing interests

The authors declare that they have no competing interests.

## Authors' contributions

RG does to the experiments and data analysis, HQ works on computational simulation and JW contributes to experimental design and manuscript writing.

All authors have read and approved the final manuscript.
